# Assessing Willingness to Engage in Risky Driving Behaviour Using Naturalistic Driving Footage: The Role of Age and Gender

**DOI:** 10.3390/ijerph181910227

**Published:** 2021-09-28

**Authors:** Petya Ventsislavova, David Crundall, Pedro Garcia-Fernandez, Candida Castro

**Affiliations:** 1 Department of Psychology, Nottingham Trent University, Nottingham NG1 4FQ, UK; david.crundall@ntu.ac.uk; 2 Electronic and Computer Sciences Department, Faculty of Sciences, University of Granada, 18071 Granada, Spain; pedrogarcia@ugr.es; 3 CIMCYC, Mind, Brain and Behaviour Research Centre, Experimental Psychology Department, Faculty of Psychology, University of Granada, 18071 Granada, Spain

**Keywords:** risk estimation, risk appraisal, decision making, age, gender, driving experience

## Abstract

Young novice drivers are more prone than older drivers to get involved in a risky driving situation. Some young drivers underestimate risk while overestimating their driving abilities, increasing the likelihood of engaging in risky behaviour. Age and inexperience both influence risk estimation, though it is not clear which of these variables is more important. Can drivers’ willingness to engage in risky behaviour be assessed in a similar way to hazard perception skill using video-based risky situations? The aim of the current study was to assess whether a video-based tool could measure the willingness to participate in risky driving situations and whether it can distinguish between different types of risky driving scenarios across gender and driver age groups. We also explored the moderating effect of age and gender on drivers’ experience in relation to the risky manoeuvres and participants’ willingness to engage in risky situations. Participants were presented with naturalistic videos from the perspective of the driver that contained active risky situations (result of driver’s own actions) and were asked to make a decision regarding a potential action (to overtake a bus/bicycle or pass through an amber light) and whether they would accelerate at this point. Participants reported that they were more willing to accelerate and overtake cyclists and buses and less willing to pass a light in amber. Young drivers were more willing to both engage in the risky behaviours and accelerate than older drivers, with young males reporting higher scores than the other groups. Gender differences were observed, with males being more prone to overtake and pass through a light in amber than females; however, this difference was not observed for the intention to accelerate. All the above effects remained when we tested the impact of experience on decision making while controlling for age and gender, although driving experience was no longer significant. These results demonstrate that drivers’ intention to assume risk can indeed be measured in a similar video-based methodology to that used by hazard perception tests. The findings raise the possibility of assessing and training drivers on a wider range of safety-related behaviours.

## 1. Introduction

Road traffic collisions are responsible for approximately 1.35 million deaths per year across the world [1] and are the primary cause of death for young people aged between 5 and 29 years old [2]. In Europe, 25,300 people lost their lives in traffic collisions, of which 14% were between 18 and 24 years old. These numbers are worrying considering that this cohort comprises only 8% of the total EU population. In the UK, young drivers aged between 17 and 19 are involved in 9% of fatal and serious crashes where they are the driver [3], even though they only represent 1.5% of UK licence holders [4]. Young people aged between 16 and 19 are three times more likely to get involved in a fatal car crash than drivers aged 40–49 [5]. Specifically, young male drivers are those who represent the highest numbers of road deaths when compared to their female peers [3]. Research shows that youth and inexperience is responsible for crashes in a significant proportion of young drivers [6]. Some of these crashes are due to a lack of fully developed skills (such as hazard perception; [7,8]), though other crashes are due to risky driving behaviours [9]. The willingness of a driver to engage in risky driving behaviour depends on their perception of the danger involved in performing such actions (risk estimation) and the perceived reward vs. costs that may be gained (e.g., peer respect, arriving sooner at a destination). The intention to engage in risky behaviour may also be moderated by drivers’ positive or negative attitudes towards taking risks (risk propensity vs. risk aversion, [10]).

Risk estimation is defined as the subjective judgement of the likelihood of suffering negative consequences due to being involved in a potentially risky situation [11]. The willingness to engage in a risky driving behaviour, however, is the action to adjust the driving behaviour to the degree of risk drivers are willing to take [12]. If the perceived risk falls below the subjectively accepted degree of risk, people are more prone to engage in risky actions or make risky decisions [13]. Some studies suggest a negative correlation between perceived risk and willingness to engage in risky driving behaviour (i.e., the more dangerous or likely a negative outcome is, the less likely a driver will be to engage in that action, [14]), while others have found a positive relationship between the two [15]. Several theories support the notion of a negative correlation between risk estimation and the willingness to engage in risky behaviour such as The Heath Believe Model [16], the Theory of Reasoned Action [17] and the Theory of Planned Behaviour [18], and there are empirical data to support this hypothesis [18,19,20,21,22]. In contrast, others have found a positive relationship between risk estimation and willingness to engage [23,24], and this relationship has been supported by the dual processes approach [25], which favours the use of fuzzy, gist-based memory representations in decision making as opposed to more verbatim representations. Mills et al. [26] rationalize the contradictory findings of these studies with the observation that negative correlations between perceived risk and willingness to engage in risky driving behaviours are often found in studies that involve immediate holistic or gist processing of a situation, while positive correlations are found in studies that allow participants time to compare the positive and negative outcomes. While both processes might occur across the range of potential driving scenarios, the dynamic nature of driving is likely to evoke an immediate ‘gut’ response to the level of risk. Thus, we might expect more instances of perceived risk negatively correlating with willingness to engage.

Young drivers who have been involved in road crashes may fail to perceive risk in a holistic way [27,28,29] and focus on specific hazards rather than the overall level of risk that a certain situation may involve [30]. They may also be slower at detecting common hazardous situations [8,31,32], which may influence risk-taking behaviours through poor understanding of on-road dangers [33]. Young drivers have been found to be less likely to rate speeding as high risk than older drivers [34,35]. Even when a specific driving behaviour is perceived as high risk, some young drivers may still engage in it [34,36]. The reason for this could be due to a recurrent lack of negative feedback (e.g., crashes, penalties; [37]). Therefore, unrealistic risk estimation could be seen as the result of the lack of negative consequences, which in turn could increase the probability of engaging in risky behaviours [38]. If there are no direct negative consequences, young drivers may wrongly learn that this type of driving behaviour is acceptable, increasing their willingness to accept risk [38,39]. The lack of adverse consequences will influence the judgement of the risk this situation involves and on the willingness to engage in risky behaviours associated to it [40]. Those young drivers that have not suffered negative consequences as a result of their risky behaviour can report lower levels of perceived risk and tend to repeat these behaviours. This pattern of behaviour supports the protective relation with risk estimation as noted by Milles et al. [26], where risk estimation is negatively correlated with risk taking. As a result, underestimating risk is associated with a higher propensity to engage in risky behaviour [40,41]. Some studies have demonstrated that the majority of young drivers tend to underestimate risk and engage in risky driving even after being involved in a car crash [42]. Although these drivers were observed to be more cautious while driving during the first two months after the collision, this cautiousness was no longer observed after the third month.

A well-known theory that has focused on the underlying process of decision making in driving is the Risk Homeostasis Theory [13]. This theory explains that the decision to engage in a particular behaviour is influenced by the comparison of perceived risk associated with that behaviour and the ideal level of risk (i.e., arousal) that the driver prefers (which may change over time and circumstances). If the perceived levels of risk are higher than their preferred level, drivers modify their behaviour to bring their current risk levels within their personal acceptable boundaries. Therefore, it is expected that drivers will drive more cautiously on dangerous roads providing that they perceive the road to be dangerous, and that their level of required arousal is lower than that provided by the associated risk. This theory also implies that if the level of risk or arousal is lower than their preferred level, they will modify their behaviour to increase arousal, which could include engaging in risky behaviours such as distraction via mobile devices or an increase in speed. However, a more recent and comprehensive approach to risk estimation is the Risk Allostasis Theory [43]. According to this theory, individuals choose a preferred range of risk in which they operate and adapt their behaviour according to the limits of this range. This theory offers a more flexible approach than the Risk Homeostasis Theory as the range of preferred risk can be altered according to the individual’s motivations and perceptions of their own capability. Current and long-term motivational factors can modify the preferred range of risk and, consequently, their behaviour will be adjusted and maintained within this range.

Emotions have also been linked to the willingness to engage in risky driving behaviour [44,45,46]. The appraisal tendency framework suggests that fear enhances drivers to perceive higher risk levels, while anger was found to induce more optimistic risk perception and poor driving behaviour [45,47,48], potentially increasing the likelihood of collisions [49]. It has been argued that individual control is associated with anger and influences risk perception in driving [50]. This false perception of control could make young drivers believe that they are capable of handling complex situations [51], which in turn makes them more susceptible to distractions whilst driving [52] and engaging with their mobile phones [53]. In the same line, young drivers may underestimate risk due to a reduced sense of fear [54], especially when they are not conscious about the level of risk associated to certain situations [55,56].

### 1.1. Gender

Factors such as gender, age [38,57] and lifestyle [58] have been found to contribute to risky driving behaviour. Males typically assume higher risk when compared to women [59,60], comply less with traffic norms [61] and are overrepresented in crash rates [62,63,64]. Male drivers also commit more traffic offenses than females [65,66]. It has been argued that both biological and psycho-social reasons could explain these differences. For example, the difficulty to inhibit impulsive behaviour could be one of the reasons why males are overrepresented in crash rates. In addition, gender differences in risky driving have also been defined by social expectations (in terms of gender roles) for compliance with traffic regulations [67] and risk taking [68]. In a recent study of reoffending following penalization, McDonald et al. [69] found that young male drivers were more likely to reoffend if their violation was classed as dangerous and careless driving, when compared to similar drivers penalized for other violations (e.g., seatbelt violations).

### 1.2. Age and Driving Experience

Results are contradictory in relation to the weight of importance of experience and age in drivers’ proneness to engage in risky driving. While driving skills are mainly influenced by driving experience [28,38,70], the decision to engage in risky driving seems to be more associated with age [71]. For instance, much research has identified that drivers younger than 25 have an increased willingness to engage in risky driving behaviour compared to their older counterparts [72,73,74]. However, even though the age of 25 is often quoted as an important neurodevelopmental milestone for the prefrontal cortex [75], it is unlikely that all risk taking ceases at this age. Unfortunately, there is limited research addressing the willingness to engage in risky driving behaviours across a more fine-grain range of ages, such as those in their late 20 s and early 30 s.

Some studies have discussed the impact of age and driving experience in relation to crash risk. McCartt et al. [76] reported independent effects for experience and age. They compared drivers over the age of 25 with younger novices and found differences between the two groups with younger novices reporting higher crash rates. Curry et al. [77] also observed that young drivers at a similar age but with different driving experience report different crash rates. In their study, young novice drivers at the age of 21 were involved in more road crashes than experienced drivers at the same age, but both groups reported considerably lower collisions in comparison to 17- to 20-year-old novice drivers. However, not many studies have explored the effects of age and driving experience on the proneness to engage in risky situations.

What is well known is that young male drivers tend to be more optimistic about their driving ability when compared to older male drivers, and they are more willing to engage in risky behaviour [78]. They also perceive certain traffic situations such as speeding or phone use as less risky in comparison to older drivers [34]. While there is evidence to suggest that hazard perception skills improve with experience, risk appraisal seems to be more affected by age. McKenna et al. [79] suggested that hazard perception and risk estimation could be impacted in a different way by driving experience and age; therefore, they should be studied separately. In consequence, it could also be argued for a differential effect of age and driving experience in relation to the willingness to engage in risky driving behaviour.

### 1.3. Risky Driving Manoeuvres: Overtaking and the Amber Light Dilemma Zone

Common risky behaviours are the use of mobile phones while driving [80], driving under the influence of alcohol or other drugs [34,81,82], speeding [83] and dangerous lane changes [84]. Anther common risky manoeuvre is overtaking, which can include speeding and dangerous lane changes. Overtaking has been associated with a high probability of crash risk (specifically when overtaking using a contraflow lane; [85]). Even though drivers seem to be aware of the danger of this manoeuvre [86], the type and speed of the vehicle to be overtaken may influence their willingness to engage with it. Pai [87] looked specifically at buses and cyclists and found that an overtaking manoeuvre by the longer vehicle usually results in a collision with the cyclist.

Drivers choose to engage in overtaking manoeuvres in order to reduce travel time and avoid platoons (group of vehicles that travel at close proximity matching the speed and manoeuvre of the lead vehicle; [88]). However, unsafe passing manoeuvres, that can lead to collisions, are often a result of frustration with slow traffic. To avoid delays during slow traffic, some drivers are willing to assume more risk as they overestimate their ability to complete the overtaking manoeuvre safely [89]. In consequence, they are more likely to reduce the critical gap and perform the overtaking manoeuvre [90], and even may accelerate to overtake vehicles travelling at faster speed [85]. However, Kinnear et al. [91] showed that the drivers in their study seemed to be able to control their frustration with longer platoons as they were able to resist the temptation to accelerate and overtake. In addition, their results suggested that time pressure did not significantly impact on the intention to overtake, although slow traffic did increase frustration. They examined the impact of different factors such as time pressure, speed, length of platoons and proportion of heavy goods vehicles (HGVs) on frustration and overtaking intentions, by showing participants video clips that contained a combination of all the above factors. Kinnear et al. [91] found that lower speed is associated with a greater intention to overtake, although this effect is susceptible to change in intentions with the presence of other factors such as platoon length and oncoming traffic. Kinnear et al. [91] did not report, however, whether the intention to accelerate and overtake vary in relation to specific traffic situations.

The estimation of the severity of a specific driving situation has been found to influence drivers’ decisions to accelerate [28]. Harbeck and Glendon [92] observed that the young drivers in their study showed high willingness to engage in speeding-related behaviours. Many risky behaviours are risky without the need to speed (e.g., overtaking, running an amber light), yet some drivers compound the risks inherent in the behaviour by increasing their speed while making the manoeuvre. While speeding up while overtaking or passing through a changing traffic light may be perceived as a way to lessen the risk of the base manoeuvre (i.e., the overtake is completed more quickly, or you clear the junction before opposing traffic begins to move), adding speed to an already risky situation may merely increase the danger (such as increasing collision speed in the contraflow lane, or raising the possibility of losing control through a junction).

Studies have reported that some of the most frequent risky behaviours are speeding while overtaking and when traffic signals/lights are amber. Specifically, amber lights have been deemed as highly ambiguous as they require the driver to decide whether to stop or pass through it [93,94]. When a vehicle approaches an intersection at a higher speed at the onset of yellow phase, this vehicle is trapped in the dilemma zone. Within this zone, a decision to proceed could result in a right-angle crash, whereas a decision to stop might produce a rear-end collision. The most crucial factors for making a decision to pass through amber lights seem to be the amount of distance from the yellow light, the position and the approaching speed of the vehicle [93].

The dilemma zone boundaries seem to vary across different age and gender. While young drivers were found to speed and ran through red lights more often [95], older drivers seemed more likely to pass a light in amber [96]. However, drivers older than 65 years of age are significantly less likely to get through the intersection at a closer onset yellow light distance when compared with other age groups [97]. In terms of gender, females seem more likely to find themselves in the dilemma zone than male drivers [97], although results are mixed with some studies reporting opposing findings (especially in relation to speeding through amber lights) [98]. Although gender and age clearly impact on the decision to engage in risky driving manoeuvres, results are contradicting and unclear. Similarly, neither risky overtaking nor running through amber light have been studied in relation to both age and gender for subsequent interactions or compared with each other.

### 1.4. Driving Scenarios According to the “Active” vs. “Passive” Role of the Driver

It is essential to distinguish between “active” and “passive” hazards when creating hazard perception/risky decision-making tests. The confusion surrounding these two concepts has created an important confound related to the method of risk estimation and risky decision-making scoring. This confound is especially evident with Deery and Love’s [99] study, where both active and passive hazards were included in a conventional hazard perception test. They defined active hazards to be a result of driver’s own actions and passive hazards to be caused by other road users.

Other tests that have measured risk estimation and the proneness to engage in risky driving behaviour featuring hazardous videos have been developed by McKenna and Crick [11] and Horwsill and McKenna [7]. They assessed everyday risk taking in driving behaviour by asking participants at what speed they choose to continue driving. Similarly, the Vienna Test [12,100] uses video clips to assess the willingness of drivers to take a risk. Hergovich et al. [12] studied whether the propensity to take a risk can be assessed as a personality trait via the Vienna Risk-Taking Test–Traffic. They asked participants to indicate at which point they consider that the situation has become too dangerous to proceed. The video clips used in their study required participants to make a decision in relation to different risky active situations such as overtaking manoeuvres, speed choices and intersections. In a recent study, Castro et al. [101] looked at the possibility to assess the process of hazard prediction and risky decision making independently, showing participants both active and passive hazards. They assessed young drivers and found that those participants with less experience were less able to predict upcoming hazards and more likely to make riskier decisions. Familiarity with the clips also influenced risk-taking behaviours: driving scenarios from another country (UK) evoked riskier decisions by the participants than those from their home country (Spain). The results suggested that risky decision making can be assessed in a similar way to the hazard prediction skill. Castro et al. [101], however, did not investigate differences in responses across the hazardous scenarios featured in their test (overtaking bus/bike and running through amber light) or whether gender and age impact drivers’ decision to assume risk in each of these situations.

### 1.5. The Current Study

This study aims to assess whether the willingness to engage in risky driving situations and risky decision making could be measured in a similar way to the hazard prediction skill by featuring a video-based test where videos are occluded prior to the development of the risky scenario. We assess whether the selected scenarios can differentiate between different age groups, driving experience and gender. We also assess whether risky decision making and the willingness to accelerate differ across the selected scenarios. Finally, we test the influence of age, driving experience and gender independently on the willingness to engage in risky driving situations, while controlling for the impact of age and gender on driving experience.

In order to test the above questions, we used the same clips of real traffic situations (recorded in the UK) as Castro et al. [101]. However, unlike Castro et al., the study was conducted online, and clips were presented via online software to test whether this version would provide valid results. Furthermore, Castro et al. assessed risky decision making in Spanish drivers, whereas the sample for this study was comprised by UK participants. Each clip contains a risky situation that may happen (e.g., overtaking a cyclist on a dual carriageway). The core design of the test is similar to the hazard prediction tests [102,103] with clips occluded by a black screen at a crucial decision point, in this case immediately prior to a potential risky situation. Participants are then asked to make a decision (via a rating scale) about their willingness to engage in the imminent behaviour (e.g., whether to overtake the bus/bicycle or pass through the amber light). Participants are asked to assume the role of the film-car driver (therefore, these situations can be considered active scenarios) and to make a decision on whether they will engage in performing the risky driving behaviour. Participants are also asked to rate whether they would accelerate at this point.

We predict that young drivers will accept a higher degree of risk than the older drivers. Furthermore, males in both age groups are expected to accept higher levels of risk than the females. These differences may change across the different driving scenarios, although we do not make a prediction about which risky situation will receive the highest rating scores. We also predict that age will have a moderating effect on drivers’ experience in relation to the risky manoeuvres and drivers’ willingness to engage in risky situations.

## 2. Materials and Methods

### 2.1. Design

A mixed design 2 × 2 × (3) was used for this study, with the variables of driver age (with participants split into those younger and older than 30 years), gender (male, female) and the type of risky scenario shown to participants (i.e., overtaking a bus, overtaking a cyclist, running through a light in amber). The dependent variable was participants’ ratings, which they provided after viewing 10 video clips containing a mixture of the three risky scenarios. Each participant gave two ratings for each clip on a 1 to 7 scale. The first was a rating of how likely they thought they would be to engage in the driving behaviour implied in the clip. The second rating reflected how likely they thought they would be to accelerate in that scenario. Analyses of variance and covariance (ANOVA and ANCOVA) were conducted to compare ratings across the three factors and to control for the effects of age and gender on driving experience.

### 2.2. Participants

In total, 243 participants took part in this study. Participants were all UK based and recruited via online social media platforms. In terms of age, 164 participants were younger than 30 years old (35 males; 129 females) and 79 were at the age of 30 or older (26 males; 53 females), with 18 being the minimum age. The age range oscillated between 18 and 76 years old (males 18–76; females 18–73). Experienced drivers (*n* = 154; 42 males; 112 females) were those with more than 3 years of driving experience since passing their driving test and an average mileage of 23,297 miles (37,492 kilometres) in the previous two years. Novice drivers (*n* = 89; 19 males, 70 females) had less than 3 years of driving experience and an average mileage of 7729 miles (12,438 kilometres) in the previous two years. For reference, please see Table 1.

### 2.3. Materials

#### 2.3.1. Filming and Video Editing

**Figure 1:**
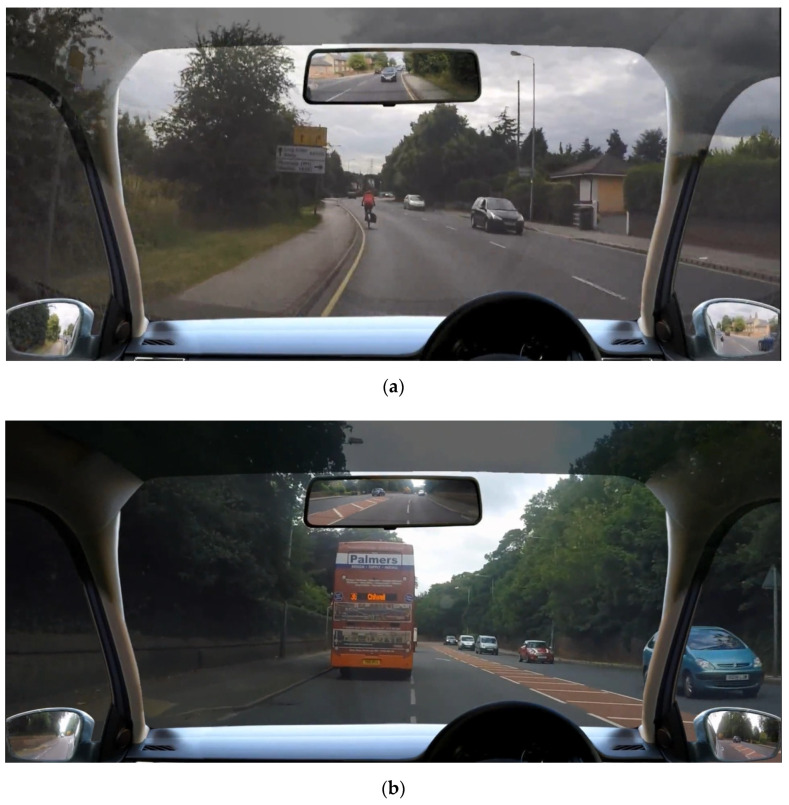

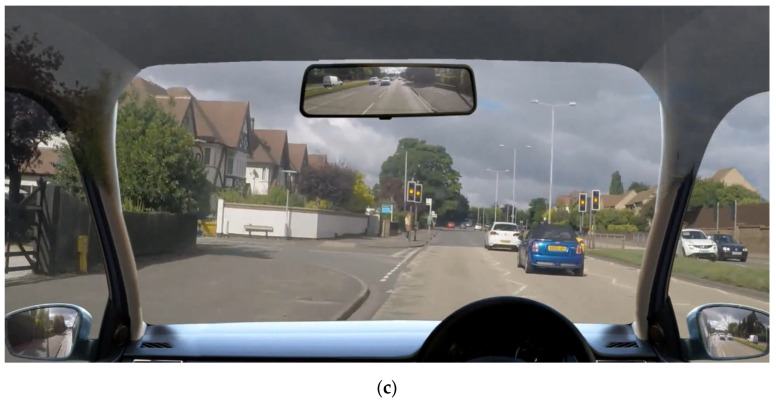
Screenshots of the final frame (prior to occlusion) of the selected risky situation: (**a**) The top panel depicts a situation with a cyclist on the left. The cyclist is further out from the camera car with oncoming traffic. The overtaking manoeuvre is considered not to be safe due to the oncoming traffic and poor visibility of the road ahead. (**b**) The middle panel shows a situation with a bus in front of the camera car. The overtaking manoeuvre is considered not to be safe, as markings in the middle should not be entered with such poor visibility of the road ahead (with red area to increase awareness of potential danger and road arrows indicating that the two lanes are about to merge in one lane). (**c**) The bottom panel depicts a situation with lights in amber. Passing through the amber lights is not safe, as the distance the camera car is travelling at will not reach the traffic lights before those turning red. The risk is greater for the pedestrian, visible on the left, who could be about to step into the road.

This study was conducted in accordance with the Declaration of Helsinki Ethical Principles for Research. The video footage was recorded during normal driving in Nottingham and suburban areas of Nottingham. The footage was filmed following the Health and Safety guidelines approved by the School of Social Sciences Ethics Committee, Nottingham Trent University (18122019). None of the risky situations were staged. Four cameras were mounted on the driving car, capturing information coming from the forward view and footage that would normally be seen in the side and rear-view mirrors (see Figure 1). All cameras were mounted via suction mounts on the outside of the windshield, right and left windows and the rear window. None of the cameras obstructed the view of the driver. GoPro HERO4 Silver Edition camcorders, recording in full high-definition format (1080 p, 16:9 ratio, medium angle setting) were used, see [103,104]. The footage from all four cameras was synchronised and edited into windscreen and mirror placeholders that were contained in a semi-transparent graphic overlay of the inside of a car. The overlay was designed to be transparent from halfway up the A-pillars, allowing for the forward view to be seen through these sections of the overlay (simulating small head movements in real life, which help focusing on a potential hazard behind an A-pillar).

#### 2.3.2. Video Clips

Ten video clips were selected for this study by a group of traffic psychologists who are considered experts in the field. The video clips contained three types of potential risky situations. Each one of the clips was designed to assess the critical interval during which drivers decide whether to stop or proceed with a possible risky manoeuvre (e.g., [93,98]). Three clips included situations where a cyclist was visible ahead of the film car in the same lane. The clips cut to black at the moment the film car had to make a decision about whether to overtake the cyclist or remain in the same position (Figure 1, top panel). Another three videos contained footage with a bus driving in front of the film car. The videos occlude at the point where the film car is close enough behind the bus to make a decision on whether to overtake (Figure 1, middle panel). Finally, four clips contained situations in which the traffic lights ahead turn to amber. The videos cut to black at the point when drivers should have noticed the changing light and then must make a decision on whether to pass through the traffic lights or stop (Figure 1, bottom panel). If participants were to be shown the full clip, they would have seen that the camera car did not perform any of the risky manoeuvres. It should be highlighted that none of the selected overtaking/amber light scenarios were safe for the driver to proceed (each scenario was judged by a group of transport psychologists who were experts in the field). While on some occasions overtaking a bus or a cyclist could be safe (e.g., the road is sufficiently clear ahead with a suitable gap with the other road user, without causing the driver to exceed the speed limit), performing this manoeuvre for the selected scenarios is considered as dangerous (see Figure 1 for a detailed explanation). Subsequently, for none of the selected scenarios was safe to accelerate (although with safe and legal overtaking, accelerating will help complete the manoeuvre quicker). The stimuli were presented via the online platform Qualtrics in a randomised order.

### 2.4. Procedure

Participants were recruited via social media platforms and invited to participate through a link to the online study. The video clips were presented through the online platform Qualtrics (accessed on 25 September 2021). Participants were instructed to use a computer screen to undertake the experiment in order to display the video clips at an adequate resolution. Those who attempted to access the study via their mobile devices were redirected to the end of the survey and invited to use the link again from a computer device. All participants were presented with information about the study and asked for consent to participate via tick box. Next, they were instructed that they were going to see 10 driving video clips, which would stop prior to a specific risky situation, after which they were asked to make a decision in relation to each one of these situations.

Participants were presented with all 10 video clips (in a randomised order) and after each clip they were asked to rate from 1 to 7 (where 1 was not at all and 7 most likely), “How likely is that you will…”, “overtake the bus?”, or “overtake the cyclist?” or “pass the amber light?”, with the exact question determined by the clip. Following a response to the first question, participants were presented with a second question: “How likely is that you accelerate at this point?” Higher ratings on either scale were interpreted as increased proneness to engage with the risky situation. After the experimental block, participants were asked to answer a brief demographic questionnaire about their driving experience, miles driven, age, etc.

## 3. Results

### 3.1. Internal Consistency

We started by calculating the Cronbach Alpha coefficient, which provided a score of 0.83. This result suggested a good internal consistency.

### 3.2. Age, Gender and Risky Situation

#### 3.2.1. “How Likely Is That You Overtake the Bus/Cyclist or Pass through the Amber Light?” (Q1)

**Figure 2:**
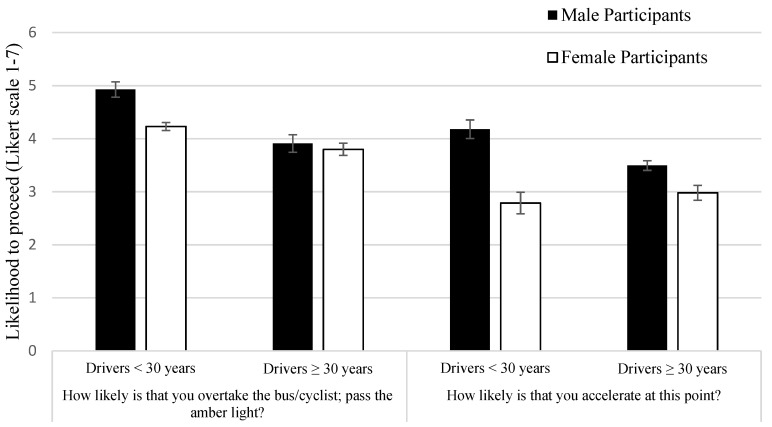
Mean ratings breakdown for gender and age across the two questions: “How likely is that you overtake the bus/cyclist; pass the amber light?” and “How likely is that you accelerate at this point?” (with standard error bars).

A 2 × 2 × (3) ANOVA comparing the ratings of the drivers by age, gender and for each risky situation was conducted. A significant main effect was found for participants’ age (F(1, 239) = 31.14, *p* < 0.001, η2 *p* = 0.12). Those participants who were younger than 30 years old reported higher ratings (M = 4.58) than those older than 30 years old (M = 3.86). Younger drivers seem to be more willing to undertake the risky behaviours in our scenarios than those drivers older than 30. Similarly, a significant main effect was found for gender (F(1, 239) = 9.43, *p* < 0.005, η2 *p* = 0.04). Males provided higher ratings (M = 4.42), being more prone to overtake/pass traffic lights in amber in comparison to women (M = 4.02). A significant interaction for age and gender (F(1, 239) = 5.07, *p* < 0.05, η2 *p* = 0.02) revealed that young males were those who were more prone to engage in risky manoeuvres in comparison to the other groups (see Figure 2).

No significant interactions were found for gender vs. type of risk situation or age vs. type of risk situation.

**Figure 3:**
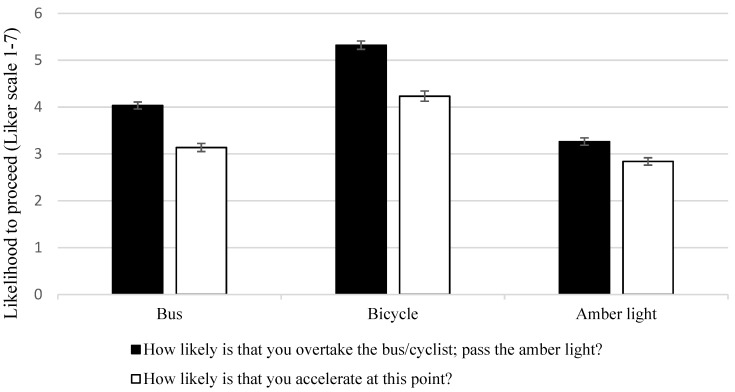
Ratings for the different type of risky scenarios across the two questions: “How likely is that you overtake the bus/cyclist; pass the amber light?” and “How likely is that you accelerate at this point?” (with standard error bars).

A significant main effect was found for the type of risk situation (F(2, 478) = 144.5, *p* < 0.001, η2 *p* = 0.377). Driving situations that included cyclists received the highest rating scores (M = 5.4), followed by those featuring a bus (M = 4.1), with the lowest ratings received for driving through an amber light (M = 3.5). Drivers seem to be more likely to proceed and overtake cyclists and less likely to pass a light in amber (Figure 3). All pairwise comparisons were significant (*p* < 001). *p*-values were adjusted for multiple comparisons with the Bonferroni correction.

#### 3.2.2. “How Likely Is That You Accelerate at this Point?” (Q2)

A 2 × 2 × (3) ANOVA comparing the ratings of the drivers by age, gender and for each risky situation was performed. The results echo the findings for the first question in relation to age. Participants’ age was significant (F(1, 241) = 36.18, *p* < 0.001, η2 *p* = 0.13) with young drivers, who were less than 30 years old, showing higher ratings and more willingness to accelerate (M = 3.84) than older drivers (M = 2.88). A significant interaction between age and gender (F(1, 241) = 7.67, *p* < 0.01, η2 *p* = 0.03) revealed that young male drivers are more likely to accelerate in comparison to the other groups (see Figure 2).

However, the main effect for gender was not replicated for this second question regarding acceleration (F(1, 239) = 2.41, *p* = 0.122, η2 *p* = 0.01). There was no significant interaction for age or gender vs. type of risk situation.

A main effect was found in relation to the type of risk situation (F(2, 478) = 68.8, *p* < 0.001, η2 *p* = 0.224). The pattern of results mirrors those found with responses to the first question. Drivers provided higher ratings and were more likely to accelerate when overtaking a cyclist (M = 4.4), followed by overtaking a bus (M = 3.3) and finally when passing traffic lights in amber (M = 3) (see Figure 2). Pairwise comparisons were all significant with levels for bus vs. cyclist (*p* < 0.001) and bus vs. amber light (*p* < 0.02). *p*-values were adjusted for multiple comparisons with the Bonferroni correction.

#### 3.2.3. Correlations between the Two Questions

Both questions correlated significantly r(243) = 0.715, *p* = 0.001, with higher proneness to engage with the risky situations translated into higher intention to accelerate. We also explored the individual correlations for each risky driving situation. The intention to overtake a bus and a bicycle correlated significantly with the intention to accelerate while performing the overtaking manoeuvre r(243) = 0.668, *p* = 0.001 and r(243) = 0.611, *p* = 0.001, respectively. A significant correlation was also observed between the intention to pass through a light in amber and accelerate r(243) = 0.842, *p* = 0.001

### 3.3. Assessment of Age and Gender on Driving Experience

#### 3.3.1. Driving Experience

We compared those drivers who were classed as experienced drivers (>three years of driving experience) with those classed as novices (<than three years of driving experience) and a main effect was found for driving experience for the first question (F(2, 239) = 10.5, *p* < 0.001, η2 *p* = 0.042). Novices (M = 4.6) were more likely to overtake/pass amber light in comparison to the experienced drivers (M = 4.1). Similarly, novices (M= 3.8) provided higher ratings than the experienced drivers for the second question (M = 3.3), (F(2, 239) = 10.9, *p* < 0.001, η2 *p* = 0.044). They were more likely to accelerate when compared to the experienced drivers. No significant interactions were observed for experience vs. type of risky situations for any of the questions.

We also observed a considerably high correlation between age and driving experience r(243) = 0.985, *p* = 0.001. Therefore, it is possible that the significant effect of experience is caused by this high correlation between the two variables. To test the effect of age on driving experience, we fitted age and gender as covariates and conducted and ANCOVA.

#### 3.3.2. Driving Experience with Age and Gender as Covariates

Both age and gender were fit as covariates to test whether there is a possible moderating effect on drivers’ experience for each one of the risky manoeuvres (overtake/amber light and accelerate). A one-way ANCOVA was conducted to compare the impact of experience whilst controlling for age and gender. Both covariates significantly impacted on the model for the first question. Higher ratings for overtaking/passing amber light were influenced by age (F(1, 239) = 18.09, *p* < 0.001, η2 *p* = 0.70) and gender (F(1, 239) = 13.13, *p* < 0.001, η2 *p* = 0.52). The effect of experience, however, was no longer significant (M = 4.2 experienced vs. M = 4.2 novices), (F(1, 239) = 0.89, *p* = 0.765, η2 *p* = 0.000). The amount of variation accounted for by the model (SSM) decreased to 271.53 (corrected model) of which experience accounted for 0.07 (previously the model accounted for 3133.16 and experience for 8.35).

Equally, higher ratings for the risky situations were influenced by age (F(1, 239) = 22.25, *p* < 0.001, η2 *p* = 0.77) and gender (F(1, 239) = 5.30, *p* < 0.05, η2 *p* = 0.20) for the second question (accelerate), and the effect of experience was no longer significant (M = 3.4 experienced vs. M = 3.4 novices) (F(1, 239) = 0.04, *p* = 0.839, η2 *p* = 0.000). The amount of variation accounted for by the model (SSM) decreased to 202.47 (corrected model) of which experience accounted for 0.05 (previously the model accounted for 6357.77 and experience for 38.28). Please see Figure 2 for a mean ratings breakdown for gender and age across the two questions.

## 4. Discussion

This study reports a new online measure that assesses the intention to engage in risky driving behaviour based on the premises of using similar methodology to the hazard prediction test. The objective of this study was to develop a test able to measure the willingness to engage in risky driving situations and decision to accelerate across different driving scenarios, and differentiate between driving groups of different age, gender and experience. We showed participants video clips occluded immediately prior to the development of three potential risky scenarios and asked them to make a decision on how to proceed for each one of the selected situations. The results suggested that an online video-based measure can successfully assess the intention to engage in risky driving. Our findings showed that the younger drivers in this study had a greater intention to engage in risky driving behaviour and decision to accelerate than the older drivers, which is supported by a number of previous studies [105,106,107,108]. Drivers under the age of 30 showed greater willingness to overtake both cyclists and buses and to pass through the amber light in comparison to those older than 30 years old. Similarly, the younger group reported higher intention to accelerate when facing the risky situations than the older group. The literature is populated with studies that have focused on the relationship between drivers under the age of 25 and risky driving, as this age groups is often overrepresented in road crashes [109]. However, the findings of this study suggest that drivers younger than 30 could be equally susceptible to risky decision making related to accelerating, overtaking and passing through amber lights.

A finding of interest was the moderating effect of age and gender on driving experience. Initially, a difference was observed between experienced and novice drivers in relation to their decision to engage in risky situations, with novices reporting higher intention to overtake, pass through amber light and accelerate than the experienced group. However, this difference was no longer present when the effectiveness of experience was tested whilst controlling for age and gender. Both age and gender significantly predicted the scores for both questions (overtake/pass through amber light and accelerate), while the effect of experience vanished. These results are important for two reasons. First, it seems that, similar to risk estimation, age has a more relevant role than experience in the willingness to engage in risky driving behaviour. Many young drivers report greater confidence in their driving skills and are more optimistic about the consequences of being involved in a risky driving situation than older drivers [13,110]. Several studies have reported that the underlying reasons for young drivers to engage in risky situations are mainly inexperience and sensation seeking, although the relationship between age and sensation seeking has been reported to be stronger (than between experience and sensation seeking) [71]. We should be cautious, however, in assuming a direct causal path between age and risky decision making without considering other factors such as experience, vehicle type or the environment. Indeed, age has a major influence on risky driving and the level of involvement in road collisions for the majority of young drivers, even when controlling for environment and type of vehicle [9]. However, the effect of experience should not be underestimated as it has been found to influence risk estimation and decision making independently of age [76]. Hence, the importance to study the impact of these variables on the intention to engage in risky driving situations independently [79].

Second, experience is often associated with hazard perception (driving skill), which is independent of the willingness to engage in risky driving behaviour [28,38,70]. Castro et al. [101] emphasised the importance of assessing hazard perception (driving skill) and risky decision making (willingness to engage in risky driving) separately to avoid confounding both variables. This is of importance, as drivers could correctly perceive a hazard and at the same time underestimate the danger this hazard poses [111,112]. Thus, drivers could make a decision to engage in a risky driving behaviour independently of the objective danger. Certainly, more experienced drivers have encountered more opportunities over time to experience risky situations against which to calibrate their risk appraisal. However, a considerable proportion of experienced drivers (especially young ones) still engage in risky driving [76]. Therefore, separating hazard perception from the intention to engage in risky driving will allow for a better understanding of these two processes and their impact on risky decision making in different driver groups of age and experience.

The gender of participants was significant for the intention to overtake/pass amber light; however, this result was not replicated for the intention to accelerate. We have already discussed the mixed nature of the results from previous studies in relation to gender and risk, e.g., [97,98], and the current findings suggest that the impact of gender is not uniform but depends on a variety of factors. The interaction between age and gender revealed that specifically younger males were those who were more prone to overtake, pass the light in amber and accelerate. Furthermore, the absence of main effect for the intention to accelerate suggests that perhaps differences between both genders settle with age for the intention to speed but remain for the intention to engage in certain risky manoeuvres. Although it has been well documented in the literature that male drivers commit more violations than females [65,113,114], future research could focus on identifying the change both male and female drivers experience in their willingness to engage in risky driving behaviour over time, and whether this change is susceptible to specific risky manoeuvres.

In relation to the traffic scenarios, the three risky situations differed significantly in terms of ratings. While participants felt significantly more comfortable with overtaking cyclists, they were less willing to overtake buses and pass through a light in amber. Similarly, participants reported a higher intention to accelerate while overtaking cyclists, followed by overtaking busses and were less likely to accelerate to pass an amber light. This was consistent for all groups independently of age and gender, and no interactions were observed between the risky scenarios and any of the other variables. Our finding that the participants in this study seem reluctant to risk running through amber lights is consistent with previous studies where drivers have reported a weak intention to run through amber lights, although it was observed that this could change depending on the context and circumstances, e.g., [115,116]. For example, the presence of cameras at different types of intersections seems to prevent drivers from running through amber/red lights [117,118], while excessive speed, short yellow signals, false perception of safety and low traffic volume seem to make them more willing to engaging with the risky manoeuvre [119].

While our participants seemed less willing to run through amber lights, they did not show the same cautious behaviour with overtaking cyclists. Road collisions caused by overtaking cyclists are not uncommon [120,121]. Overtaking collisions with cyclists are typically due to high speed [122] or narrow safety margins [123]. Furthermore, HGVs and buses overtake cyclists, even at closer proximities than vehicle drivers [124]. Basfrod et al. [125] reported that vehicle drivers in the UK hold negative attitudes towards cyclists and feel frustrated with their presence on the road. The reason for this is the perception that cyclists do not follow the traffic norms, which makes it difficult to predict their behaviour [126]. Car drivers have a competing sense of entitlement when it comes to their right to be on the road and the uneven distribution of space often puts different road users as mutual competitors. Competition for space has become a major issue in the UK with a considerable impact on vulnerable road users. Campaigns to raise awareness and road traffic norms/policies that prioritise cyclists, pedestrians and vulnerable road users will improve the conflictive interaction between cyclists and car drivers. In addition, training to adopt the perspective of the other road user could contribute for a better understanding of the global traffic situation. Such training has demonstrated success in teaching drivers to anticipate and detect dangers and avoid road collisions with other road users, especially vulnerable road users, cyclists and pedestrians [29].

This study is not without limitations. It should be noted that online studies could bring some data quality issues due to the lack of the rigorous control inherent to the conventional laboratory settings. While participants in online studies are susceptible to distraction and often multitask, studies have reported that they are still capable of providing conscientious and thoughtful answers independently of the device or context [127,128]. In addition, this online version of the study has replicated the results obtained by Castro et al. [101], who conducted their study in laboratory settings. These results indicate that online assessment could be a plausible option for future research. While studies in hazard perception have already reported successful online training interventions [8], this study presented for the first time an online assessment of the intention to assume risk, and the results seem promising.

Another limitation of the study was the lack of gender balance in our sample. Future research should feature a more balanced sample in terms of gender (e.g., equal number of men and women) to address the contradicting results across several risky decision-making studies. Moreover, marital status seems to be another relevant variable to be considered in future research. Offender drivers have been reported to be predominantly young or slightly older single (or divorced) males [129,130]. A more thorough investigation into the mediating effect of marital status could help understand the mixed results in relation to gender.

## 5. Conclusions

In conclusion, the results of the present study suggest that drivers under the age of 30 years old could also benefit from training in adapting their driving behaviour to prevent risky decision-making behaviours. It is important, however, to implement a variety of assessment methods to train each skill individually and make drivers aware of the differences between the subjective perception of their driving skills and their real ability [131]. Furthermore, these assessment methods should also contain active hazard awareness training featuring hazardous scenarios that mimic risky driving (as a result of the driver’s own actions) to train young drivers in successfully balancing the task demands and become more aware of wrong risk calibration. The advance in technology with computer-generated imagery (CGI) could make the creation of such scenarios possible without exposing drivers to real risk [31]. Gaining more insight of the level of risk each age group is willing to assume will help adapting training according to their specific needs. Moreover, learner driver training does not focus specifically on creating awareness of wrong calibration. Young drivers who have recently obtained their driving licences abandon training shortly after passing their tests and, after gaining some experience, assume they have control over the driving situation. Therefore, it is essential to create awareness of the different skills required to become a safe driver and continue stimulating learning and training even after passing the driving test.

## Figures and Tables

**Table 1:** Descriptive statistics for all participants across age, gender and driving experience.

	*n* Total = 243							
**Age**	**Drivers under 30 Years Old** ***n* = 164**				**Drivers Who Are at the Age of 30 or Older** ***n* = 79**			
	Men *n* = 35		Women *n* = 129		Men *n* = 26		Women *n* = 53	
	Mean	Standard Deviation	Mean	Standard Deviation	Mean	Standard Deviation	Mean	Standard Deviation
Age	21.8	2.6	22	2.9	50.8	11.8	46.1	10.6
Miles driven in the last 2 years	17,385.7	15,298.6	11,385	11,566	35,000	24,147.9	24,311	30,264
Months since driving test	39.7	27.6	42.6	31.4	406.2	143.3	301.7	126.6
Collisions since driving test	0.4	0.5	0.3	0.7	2.2	2.9	1.1	1.2
**Driving Experience**	**Novice drivers *n* = 89** **(Less than 3 years of driving experience)**				**Experienced drivers *n* = 154** **(More than 3 years of driving experience)**			
	Men *n* = 19		Women *n* = 70		Men *n* = 42		Women *n* = 112	
	Mean	Standard Deviation	Mean	Standard Deviation	Mean	Standard Deviation	Mean	Standard Deviation
Age	20.3	1.4	21	2.6	40.4	16.4	34	13.7
Miles driven in the last 2 years	12,405	11,019	6459.7	6103.6	30,543	22,456	20,580	23,224
Months since driving test	21	8.6	21.9	9.4	275	203.5	178.1	147.8
Collisions since driving test	0.3	0.5	0.3	0.7	1.6	2.4	0.8	1

## Data Availability

Publicly available datasets were analysed in this study. This data can be found here: https://osf.io/cyfdu/ (accessed on 23 September 2021).
